# Laser-guided rescue: endoscopic removal of complex esophageal foreign bodies

**DOI:** 10.1055/a-2658-0099

**Published:** 2025-08-08

**Authors:** Priscilla Lopez, Mohan Ramchandani, Sundeep Lakhtakia, Krithi Krishna Koduri, Aniruddha Pratap Singh, Pramod Reddy, D. Nageshwar Reddy

**Affiliations:** 1 78470 Gastroenterology, Asian Institute of Gastroenterology, Hyderabad, India

About 10%–20% of esophageal foreign bodies require intervention 1 . Dentures are particularly difficult to remove due to their size, sharp edges, and metal parts 2 . While endoscopy is often effective, some cases need surgery 3 . We report two cases of successful laser-assisted removal of impacted foreign bodies.

**Case 1** . A 35-year-old man with alcohol use disorder accidentally swallowed his denture. After failed removal attempts elsewhere, he was referred to our center. Computed tomography (CT) showed a radiopaque object at the D3 level ( Fig. 1 ). Endoscopy confirmed an impacted three-tooth denture with a sharp metallic edge, deeply embedded in the esophageal wall, with a contained perforation. Given the failure of conventional techniques, the patient was intubated and endoscopy-guided laser lithotripsy was used for fragmentation.

**Fig. 1:**
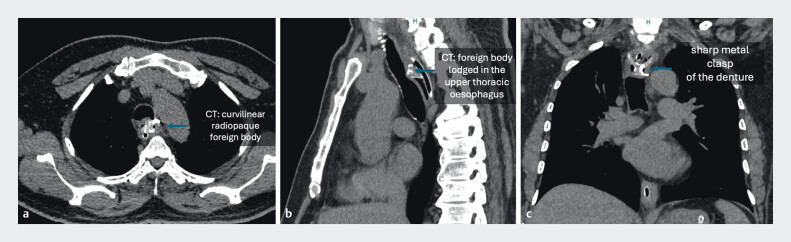
Computed tomography images. **a–c** A well-defined curvilinear radiopaque density measuring 3.2 × 6.2 cm was noted in the upper thoracic esophagus at the level of the D3 vertebral body with associated short segment circumferential wall thickening (9 mm).

Laser fiber (LightTrail Reusable 365 µm; Boston Scientific, Galway, Ireland) was preloaded onto a catheter (One Action Stent Introduction System [OASIS] internal catheter, 6 Fr; Cook Medical, Bloomington, Indiana, USA), compatible with the 2.8-mm channel ( Fig. 2 ). The laser source was a 360-nm Lumenis VersaPulse holmium laser (Boston Scientific, Marlborough, Massachusetts, USA), with settings of 9.6 W, Frequency 8 Hz, Energy 1200 mJ. This allowed precise disintegration. The narrowest section of the denture was cut ( Fig. 3 ), along with the metallic wire and acrylic resin. The entire procedure was completed in 15 minutes without collateral damage. The fragments were extracted with a snare ( Video 1 ). The decubitus ulcer-induced perforation was closed using the loop-and-clip technique.

**Fig. 2:**
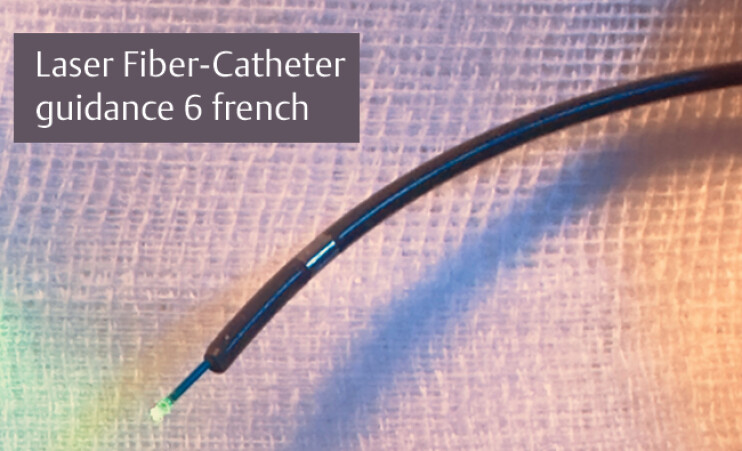
Laser fiber (LightTrail Reusable 365 µm; Boston Scientific, Galway, Ireland) was preloaded onto a catheter (One Action Stent Introduction System [OASIS] internal catheter, 6 Fr, 203 cm; Cook Medical, Bloomington, Indiana, USA).

**Fig. 3:**
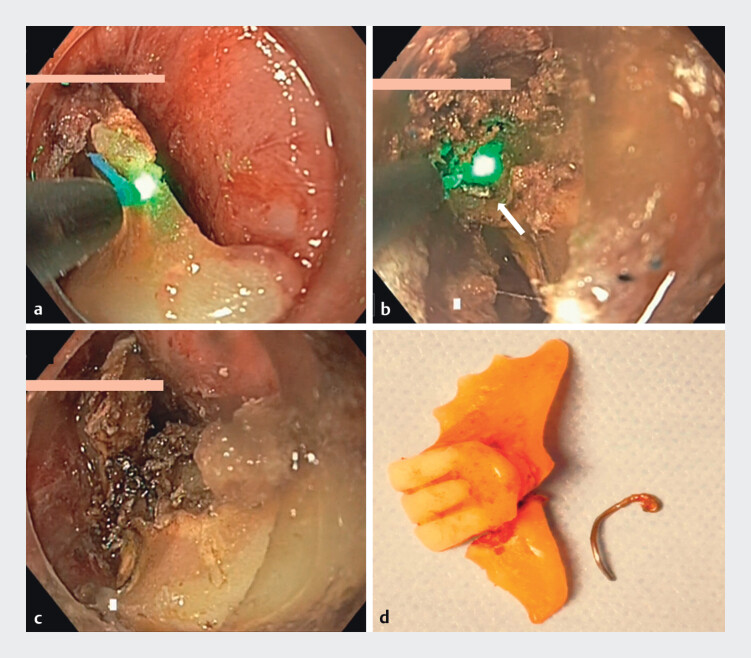
Case 1. **a** Laser-guided fragmentation of the denture. **b** Sectioning of the metallic wire. **c** Denture after complete fragmentation. **d** Retrieved denture fragments after removal.

Laser-assisted endoscopic fragmentation enabled successful removal of impacted esophageal foreign bodies and facilitated endoscopic closure, offering a safe alternative to surgery in complex cases.Video 1

**Case 2** . A 67-year-old patient presented with dysphagia after eating chicken. CT revealed a foreign body in the upper esophagus. As in the previous case, retrieval failed. The bone was fragmented using the same laser technique without complications ( Fig. 4 ).

**Fig. 4:**
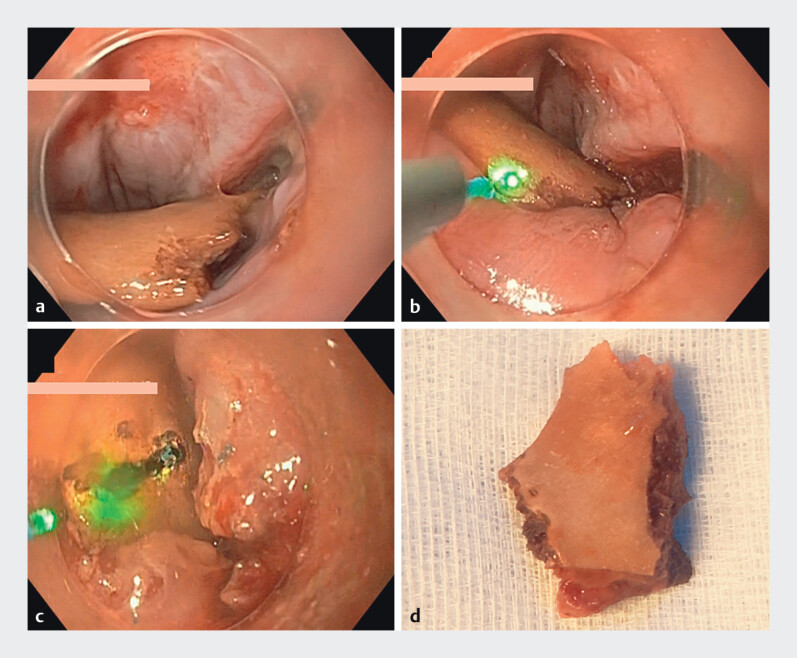
Case 2. **a** Impacted chicken bone. **b, c** Laser-guided fragmentation of the bone in the upper esophagus. **d** Retrieved bone fragment after removal.

Post-procedure assessments showed no leakage. Patients received prophylactic antibiotics, started oral intake the next day, and were discharged after 3 days, without complications.

Laser-assisted fragmentation is a safe and effective alternative for managing complex esophageal foreign bodies 4 5 , reducing surgery and enabling endoscopic closure in cases of perforation.

Endoscopy_UCTN_Code_TTT_1AO_2AL

## Notes

Endoscopy E-Videos https://eref.thieme.de/e-videos
*E-Videos* is an open access online section of the journal *Endoscopy* , reporting on interesting cases and new techniques in gastroenterological endoscopy. All papers include a high-quality video and are published with a Creative Commons CC-BY license. Endoscopy E-Videos qualify for HINARI discounts and waivers and eligibility is automatically checked during the submission process. We grant 100% waivers to articles whose corresponding authors are based in Group A countries and 50% waivers to those who are based in Group B countries as classified by Research4Life (see: https://www.research4life.org/access/eligibility/ ). This section has its own submission website at https://mc.manuscriptcentral.com/e-videos .

## References

[1] Birk M Bauerfeind P Deprez PH Removal of foreign bodies in the upper gastrointestinal tract in adults: European Society of Gastrointestinal Endoscopy (ESGE) Clinical Guideline Endoscopy 2016 48 489 496 10.1055/s-0042-100456 26862844

[2] Mughal Z Charlton AR Dwivedi R Impacted denture in the oesophagus: review of the literature and its management BMJ Case Rep 2019 12 e229655 10.1136/bcr-2019-229655 PMC6827774 31653620

[3] Singh P Singh A Kant P An impacted denture in the oesophagus – an endoscopic or a surgical emergency – a case report J Clin Diagn Res 2013 7 919 920 10.7860/JCDR/2013/5337.2976 23814744 PMC3681071

[4] Yang Z Qin S Li X Esophageal foreign body removal under holmium laser-assisted gastroscope: a case report Front Surg 2023 10 1.09416E6 10.3389/fsurg.2023.1094160 PMC9886868 36733890

[5] Vishnu Swaroop Reddy N Shekhar S Sharma M Holmium:YAG laser-assisted removal of a lamb bone impacted in the upper oesophagus Indian J Otolaryngol Head Neck Surg 2023 75 2338 2341 10.1007/s12070-023-03642-3 37636659 PMC10447732

